# Analysis of Post-Processed Pseudorange-Based Point Positioning with Different Data Sources for the Current Galileo Constellations

**DOI:** 10.3390/s24082472

**Published:** 2024-04-12

**Authors:** Jiantao Zhang, Weiwei Li

**Affiliations:** College of Geodesy and Geomatics, Shandong University of Science and Technology, Qingdao 266590, China; 202182020024@sdust.edu.cn

**Keywords:** GNSS, Galileo, GPS, single point positioning (SPP), different data sources of broadcast ephemeris, positioning performance

## Abstract

The Galileo satellite navigation system now provides initial services. With further satellite launches, the performance of Galileo will gradually improve, and new services will be introduced. This study aims to provide a comprehensive analysis of Galileo Single Point Positioning (SPP) using different broadcast ephemeris data sources. This study investigates the completeness of Galileo navigation message records from different institutions. The results show that IGS provides the best completeness across different data sources (ECR > 70%), while IGN exhibits the lowest completeness. Analyze the proportions of different data sources within the Galileo navigation message in the broadcast ephemeris files provided by IGS during the study period. The proportions of FNAV_258, INAV_513, INAV_516, and INAV_517 during the study period are 25.83%, 24.76%, 23.61%, and 25.80%, respectively, suggesting better data completeness for FNAV_258 and INAV_517 and poorer completeness for INAV_513 and INAV_516. Finally, this study explores SPP solutions for GPS and Galileo systems using different data sources. The results indicate that a higher ECR corresponds to better positioning performance. Although GPS exhibits smaller error fluctuations and smoother positioning results, Galileo’s SPP positioning accuracy surpasses that of GPS. The introduction of dual-frequency observations effectively reduces data dispersion and enhances vertical positioning accuracy.

## 1. Introduction

Over the past few decades, the Global Positioning System (GPS) has found extensive applications in various fields, including marine, terrestrial, and aviation domains. These applications encompass network-based communication, time synchronization in power systems, as well as oceanic measurements, terrestrial surveys, and monitoring of crustal movements in the field of mapping [1,2]. With its advantages of high precision, global coverage, all-weather availability, and user-friendly operation, GPS has attracted users from virtually all sectors worldwide that require navigation and positioning. Different countries have launched their own satellite navigation systems with the aim of achieving independent and autonomous satellite-based positioning capabilities. For instance, Russia has introduced the GLONASS system based on Frequency Division Multiple Access (FDMA) technology, the European Union has launched the Galileo system based on Code Division Multiple Access (CDMA) technology, and China has introduced the BeiDou Satellite Navigation System (BDS) based on CDMA technology. This signifies the onset of a new era in global satellite navigation services. The European satellite navigation system Galileo was officially declared initially operational on 15 December 2016, marking the commencement of Galileo’s initial services. Galileo Initial Services represent the first step towards its full operational capability. Upon full operational capability, the Galileo constellation will comprise 24 satellites positioned in the Medium Earth Orbit (MEO) at an altitude of 23,222 km. The eight primary satellites will be distributed across three orbital planes, and each will be inclined at an angle of 56 degrees to the equator. These satellites will be evenly spread around each orbital plane, completing an orbit around the Earth approximately every 14 h. Additionally, there will be two spare satellites in each orbital plane to account for any operational satellite failures [3]. As of March 2024, the Galileo system is moving into the Full Operational Capability (FOC) phase. A total of 28 satellites have been launched (excluding 2 experimental satellites), consisting of 23 operational satellites and 5 non-operational satellites [4]. These satellites transmit navigation signals on five frequencies: E1, E5a, E5b, E5, and E6.

Pseudorange-based SPP, as one of the fundamental modes of Global Navigation Satellite Systems (GNSS), is widely adopted across various domains due to its advantages, such as simple operation, extensive coverage, rapid positioning, and relatively high robustness. SPP relies on the utilization of lower-precision pseudorange observations and calculated receiver position and velocity from broadcast ephemeris data. Then corrections such as ionospheric delay and tropospheric delay are applied, but due to the inherent lower accuracy of broadcast ephemeris data, which is significantly below the precision of precise ephemerides and precise clock bias products, SPP typically achieves meter-level positioning accuracy [5,6,7,8]. 

Since the Galileo satellite navigation system began to provide initial services, numerous scholars have started to investigate the SPP positioning performance of the Galileo system. In 2012, the deployment of four In-Orbit Validation (IOV) Galileo satellites endowed the Galileo system with initial positioning capabilities. An evaluation of Galileo SPP with four IOV satellites was conducted, revealing comparable accuracy to GPS SPP with four selected satellites. However, it was noted that the availability of all four IOV satellites was limited both in terms of time and observing sites [9]. With the increasing number of satellites in recent years, the positioning accuracy of SPP has reached approximately 2–3 m in the horizontal component. In the vertical component, the statistical accuracy ranges from 3–4 m (using the E1 frequency) to 5–7 m (using the E5a, E5b, and E5(ab) frequencies) [3]. 

To enhance the accuracy of the SPP, many researchers have focused on optimizing the mathematical model. In terms of mathematical modeling, employing weighted least squares based on satellite elevation cutoff angles has significantly improved positioning accuracy. Furthermore, an iterative least squares method using User Equivalent Range Error (UERE) as weights has raised the accuracy of single-frequency SPP for four individual systems (GPS, GLONASS, BDS, and Galileo). This method yielded E/N/U components of 1/3/1.5 m, 1.5/3/1.5 m, and 2.5/6/2.5 m accuracy improvements for GPS/GLONASS/BDS/Galileo, respectively [10,11]. In the context of error correction models, ionospheric delay remains a significant source of error for single-frequency SPP. Simultaneously, single-frequency receivers can only mitigate the impact of ionospheric delay through various models. Such as the Klobuchar model for GPS [12], the BDGIM model for BDS-3, and the Nequick-G model for Galileo.

Furthermore, the integration of multiple GNSS systems is a promising approach to enhancing SPP performance, benefiting from an increased number of satellites and improved satellite geometry. In addition to accuracy enhancement, the combination of multiple systems can also enhance the reliability and availability of SPP positioning in challenging environments with significant obstructions, such as urban canyons, mountainous regions, and forests [13]. The integrated SPP using GPS and four Galileo IOV satellites can improve the positioning accuracy of the horizontal and vertical components by 2% and 10%, respectively [9]. A comprehensive assessment of SPP performance was conducted for GPS, GLONASS, BDS-2, and Galileo (utilizing three IOV satellites). The findings indicate that, compared to the integrated GPS/GLONASS SPP, the integrated GPS/GLONASS/BDS-2 SPP yields a 6% improvement in positioning accuracy in all east, north, and up directions. Moreover, with the introduction of Galileo observations, an additional enhancement of approximately 2% in accuracy is achievable in these three directions [14]. 

Nowadays, the widespread adoption of smartphones and mobile devices has led to remarkable growth in location-based services [15]. An analysis of SPP performance was conducted using raw dual-frequency measurements obtained from a Xiaomi Mi8 smartphone for both GPS and Galileo. Solely utilizing single-frequency GPS SPP resulted in positioning accuracies of approximately 2.2 m in the north direction and 1.7 m in the east direction. However, the accuracy improvement achieved with GPS/Galileo dual-frequency SPP solutions in the horizontal component reached 40–45%, surpassing the accuracy of single-frequency GPS solutions [16]. A comparison of GPS/Galileo dual-frequency SPP performance was conducted on recent Android smartphones, namely the Xiaomi Mi8, Xiaomi Mi9, and Huawei P30. The derived results substantiated a comparable level of positional accuracy between the Huawei P30 and Xiaomi Mi8 (4.46 and 4.56 m, respectively), both of which outperformed the Xiaomi Mi9 (7.26 m) in terms of horizontal accuracy. Concerning vertical positional accuracy, the SPP solution with Huawei P30 exhibited the best performance, with a statistical accuracy of 7.46 m, as contrasted with values of 8.56 m for Xiaomi Mi8 and 11.49 m for Xiaomi Mi9 smartphones [17]. 

In summary, existing studies have analyzed the performance of SPP in the Galileo navigation system. Many scholars have achieved significant results in improving the accuracy of SPP in the Galileo navigation system by starting with error models and integrating multiple satellite systems. However, the impact of broadcast ephemeris files on the SPP of the Galileo navigation system has been overlooked, especially considering that there are different data sources for the navigation message of the Galileo navigation system in broadcast ephemeris files and the effects of different data sources on the Galileo SPP are unknown. Broadcast ephemeris files provided by different institutions have varying degrees of differences, and the completeness rates of different data sources for the navigation message of the Galileo navigation system in broadcast ephemeris files provided by these institutions are still unknown. Additionally, as the Galileo navigation system continues to improve, there may be differences between previous analyses of the SPP performance of the Galileo navigation system and the current situation. Therefore, to address these gaps, this paper starts with conventional analysis, analyzing the number of visible satellites and position dilution of precision (PDOP) values for Galileo and comparing them with GPS. Subsequently, the completeness rates of different data sources provided by five institutions: Deutsches Zentrum für Luft- und Raumfahrt (DLR), Geodetic Observatory Pecný (GOP), Institut Geographique National (IGN), Internation GNSS Service (IGS), and WRD in their broadcast ephemeris files during the study period are statistically evaluated. Based on this, the appropriate institution is selected, and single-frequency SPP for GPS and Galileo with different data sources is solved under the condition of a 7-degree elevation cutoff angle. This analysis involves horizontal and vertical components, aiming to compare the performance of Galileo SPP using different data sources with respect to GPS. Finally, a comparative analysis is conducted on dual-frequency GPS SPP and Galileo SPP with different data sources, comparing the performance of Galileo SPP using different data sources with respect to GPS and highlighting improvements relative to single-frequency SPP.

## 2. Methodology of Galileo SPP

### 2.1. Pseudorange Observation Equations

Pseudorange measurement is obtained by determining the propagation time of satellite signals from transmission to reception and multiplying it by the speed of light to calculate the distance between the satellite and the receiver. For a specific satellite–receiver link, the simplified equations of pseudorange observations P can be described as:

(1) $$P_{r}^{s}=\rho_{r}^{s}+I_{r,j}^{s}+t_{r}-t^{s}+d_{r,j}+d_{j}^{s}$$

where s and r denote satellite and receiver ID; j denotes frequency band; $t_{r}$ and $t^{s}$ represent receiver and satellite clock offsets; $I_{r,*}^{s}$ is the ionospheric delay error;$\;d_{r,*}$ and $d_{*}^{s}$ denote receiver and satellite uncalibrated code-specific hardware biases. Moreover, $\rho_{r}^{s}$ denotes the geometric distance with the corrections of the relativistic effect, the Sagnac effect, and the tropospheric delay between the satellite and receiver.

### 2.2. Single-Frequency SPP

Taking the pseudorange observation on the first frequency as an example, linearizing Equation (1) yields:

(2) $$p_{r,1}^{s}=u_{r}^{s}\cdot x+c\delta {\bar{t}}_{r}+\varepsilon_{r,1}^{s}$$

where $p_{r,1}^{s}$ represents the computed geometric distance value minus the pseudorange observation; $u_{r}^{s}$ represents the unit vector of the component from the receiver to the satellite; x represents the increment in position relative to the previous epoch position of the receiver; c represents the speed of light in a vacuum; $\delta {\bar{t}}_{r}$ represents the reparameterized receiver clock offset that absorbs the receiver-specific pseudorange hardware delay; $\varepsilon_{r,1}^{s}$ represents unmodeled errors.

### 2.3. Dual-Frequency SPP and Triple-Frequency SPP

For dual-frequency SPP, the linearized ionospheric-free (IF) combination pseudorange observation can be expressed as:

(3) $$p_{r,IF}^{s}=u_{r}^{s}\cdot x+c\delta {\bar{t}}_{r}+\varepsilon_{r,IF}^{s}$$

with:

(4) $$\left\{\begin{array}{l} p_{r,\mathrm{IF}}^{s}=\alpha_{12}\cdot p_{r,1}^{s}+\beta_{12}\cdot p_{r,2}^{s}\\ \delta {\tilde{t}}_{r}=\delta t_{r}+\left(\alpha_{12}\cdot d_{r,1}+\beta_{12}\cdot d_{r,2}\right)\\ \varepsilon_{r,\mathrm{IF}}^{s}=\alpha_{12}\cdot \varepsilon_{r,1}^{s}+\beta_{12}\cdot \varepsilon_{r,2}^{s} \end{array}\right.$$

where $p_{r,\mathrm{IF}}^{s}$ represents the computed geometric distance of the IF combination minus the pseudorange observation;$\;\alpha_{12}$ and $\beta_{12}$ denote multiplier factors for IF combination.

For a specific satellite–receiver link [18,19], the simplified equations of triple-frequency pseudorange observations $p_{i}\left(i=1,2,3\right)$ can be described as:

(5) $$\left\{\begin{array}{l} p_{r,1}^{s}=\rho_{r}^{s}+I_{r,1}^{s}+t_{r}-t^{s}+d_{r,1}+d_{1}^{s}\\ p_{r,2}^{s}=\rho_{r}^{s}+\gamma_{2}I_{r,1}^{s}+t_{r}-t^{s}+d_{r,2}+d_{2}^{s}\\ p_{r,3}^{s}=\rho_{r}^{s}+\gamma_{3}I_{r,1}^{s}+t_{r}-t^{s}+d_{r,3}+d_{3}^{s} \end{array}\right.$$

where $\gamma_{*}=f_{1}^{2}/f_{*}^{2}$ (with f the carrier frequency) is the ionospheric amplification factor.

To be concise, the following notations are predefined:

(6) $$\left\{\begin{array}{l} \alpha_{ij}=\frac{f_{i}^{2}}{f_{i}^{2}-f_{j}^{2}},\beta_{ij}=-\frac{f_{j}^{2}}{f_{i}^{2}-f_{j}^{2}},i\neq j\\ {\mathrm{DSB}}_{ij}^{s}=d_{i}^{s}-d_{j}^{s},{\mathrm{DSB}}_{r,ij}=d_{r,i}-d_{r,j}\\ \mathrm{IF}\left(d_{i}^{s},d_{j}^{s}\right)=\alpha_{ij}\cdot d_{i}^{s}+\beta_{ij}\cdot d_{j}^{s},\mathrm{IF}\left(d_{r,i},d_{r,j}\right)=\alpha_{ij}\cdot d_{r,i}+\beta_{ij}\cdot d_{r,j} \end{array}\right.$$

where ${\mathrm{DSB}}_{r,ij}$ and $DSB_{ij}^{s}$ are receiver and satellite code-specific differential signal bias (DSB) between $P_{r,i}^{s}$ and $P_{r,j}^{s}$, which are also known as differential code bias (DCB); IF represents the operator of IF combination; $\mathrm{IF}\left(d_{r,i},d_{r,j}\right)$ and $\mathrm{IF}\left(d_{i}^{s},d_{j}^{s}\right)$ denote receiver and satellite code-specific IF signal bias (IFSB) [20]. 

For Galileo, two different clock polynomials are provided within the navigation message, which are FNAV (Free accessible Navigation) and INAV (Integrity Navigation), depending on different IF pseudorange combinations, i.e., FNAV: E5a–E1 (1176.45–1575.42 MHz), INAV: E5b–E1 (1207.40–1575.42 MHz). Thus, the broadcast satellite clock offsets can be represented as:

(7) $$\left\{\begin{array}{l} t_{\mathrm{IF}12}^{s}=t^{s}-\mathrm{IF}\left(d_{1}^{s},d_{2}^{s}\right)\\ t_{\mathrm{IF}13}^{s}=t^{s}-\mathrm{IF}\left(d_{1}^{s},d_{3}^{s}\right) \end{array}\right.$$

where $t_{\mathrm{IF}\;12}^{s}$ is estimated based on E1/E5a IF pseudorange observables, while $t_{\mathrm{IF}\;13}^{s}$ is based on E1/E5b IF pseudorange observables.

### 2.4. Correction of Broadcast Group Delay

Broadcast Group Delay (BGD) is one of the Galileo broadcast parameters commonly employed to compensate for the inter-frequency biases of satellites for single-frequency users. Each Galileo satellite broadcasts unique BGD offsets, namely BGD (E1, E5a) and BGD (E1, E5b). According to the Galileo ICD (Interface Control Document) [21], the direct relationships between BGD parameters and DCB estimates are expressed as follows:

(8) $$\left\{\begin{array}{l} \mathrm{BGD}(E1,E5a)=-\frac{f_{E5a}^{2}}{f_{E1}^{2}-f_{E5a}^{2}}{\mathrm{DCB}}_{E1E5a}^{s}\\ \mathrm{BGD}(E1,E5b)=-\frac{f_{E5b}^{2}}{f_{E1}^{2}-f_{E5b}^{2}}{\mathrm{DCB}}_{E1E5b}^{s} \end{array}\right.$$

$\Delta t_{SV}\left(X\right)$ is the satellite time correction for the signal combination X computed by means of the time correction data retrieved from the navigation message. For single-frequency positioning, the Galileo satellites clock $\Delta t_{SV}$ can be corrected using BGD parameters or more accurate DCB estimates [22]:

(9) $$\left\{\begin{array}{l} \Delta t_{SV}(E1)=\Delta t_{\mathrm{INAV}}(E1,E5b)-\mathrm{BGD}(E1,E5b)\\ \Delta t_{SV}(E5b)=\Delta t_{\mathrm{INAV}}(E1,E5b)-{\left(\frac{f_{E1}}{f_{E5b}}\right)}^{2}\mathrm{BGD}(E1,E5b)\\ \Delta t_{SV}(E5a)=\Delta t_{\mathrm{FNAV}}(E1,E5a)-{\left(\frac{f_{E1}}{f_{E5a}}\right)}^{2}\mathrm{BGD}(E1,E5a) \end{array}\right.$$

for dual-frequency pseudorange processing, no additional correction is applied for group delay.

Due to the lack of a defined correction for BGD for E5 observations in the Galileo ICD, we use DCB estimates instead for $\Delta t_{SV}\left(E5\right)$ compensation:

(10) $$\Delta t_{SV}(E5)=\Delta t_{\mathrm{FNAV}}(E1,E5a)+\frac{f_{E5a}^{2}}{f_{E1}^{2}-f_{E5a}^{2}}{\mathrm{DCB}}_{E1E5a}^{s}+{\mathrm{DCB}}_{E1E5}^{s}$$

## 3. Datasets and Processing Strategies

### 3.1. Data

In order to test and validate Galileo’s SPP performance globally, observation data sampled every 30 s from 137 MGEX tracking stations was chosen, covering a period of 50 days from DOY (Day of Year) 32 to 81 in 2021. The corresponding broadcast ephemeris files are sampled at 10 min intervals from five data agencies: DLR, GOP, IGN, IGS, and WRD. Each broadcast ephemeris for a satellite consists of eight lines. The first line records the PRN number of the satellite, the reference epoch time of the satellite clock, the satellite clock bias (s), the satellite clock drift (s/s), and the satellite clock drift rate (s/s). The remaining seven lines contain orbital parameters, as detailed in Table 1. This includes the six parameters of the Keplerian orbit at the epoch, which are the square root of the semi-major axis (sqrt(a)), eccentricity (e), argument of perigee (omega), mean motion difference from computed value at epoch (Delta n), mean anomaly at epoch (M0), and right ascension of ascending node at epoch (OMEGA0). Additionally, there are nine parameters reflecting the perturbational effects, which are the rate of change in right ascension of the ascending node (OMEGA DOT), inclination at epoch (i0), rate of change in inclination (IDOT), cosine harmonic amplitude of the argument of latitude correction (Cuc), sine harmonic amplitude of the argument of latitude correction (Cus), cosine harmonic amplitude of the radius correction (Crc), sine harmonic amplitude of the radius correction (Crs), cosine harmonic amplitude of the inclination correction (Cic), and sine harmonic amplitude of the inclination correction (Cis). The absence of one ephemeris dataset implies the loss of the satellite clock bias, satellite clock drift, satellite clock drift rate, and all orbital parameters at the reference time.

**Figure 1:**
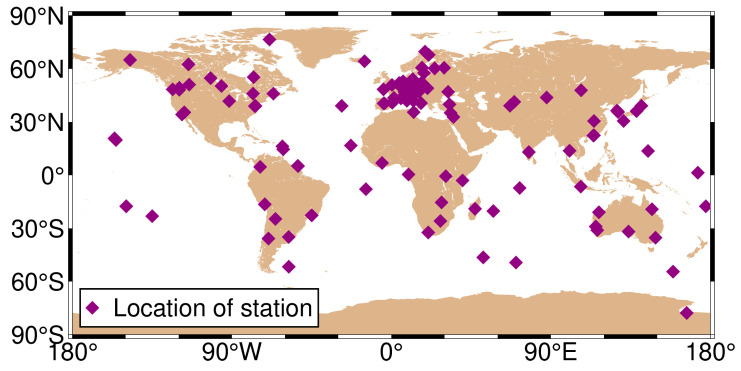
Geographical distribution of the selected 137 MGEX stations.

The period DOY 32–81 in 2021 was chosen for analysis due to reasons related to the research objectives and experimental design. The choice of a duration of 50 days was based on the need to balance statistical significance, obtain a sufficient dataset, and minimize the impact of data anomalies. Figure 1 depicts the geographical distribution of the selected stations, with purple diamond-shaped dots indicating station locations. The SPP solutions were solved using the open-source GAMP software (See: https://www.ngs.noaa.gov/gps-toolbox/GAMP/ accessed on 5 July 2023) [19]. The GAMP software is a secondary development based on RTKLIB, with many improvements such as cycle slip detection, receiver clock jump repair, and handling of GLONASS pseudorange inter-frequency biases. A simple and unified output file format has been defined for result analysis and plotting, including positioning results, number of satellites, satellite elevation angles, pseudorange and carrier phase residuals, and slant total electron content. This software not only has PPP data processing capabilities but also includes related functionalities such as SPP processing. The specific processing strategies are provided in Table 2.

### 3.2. Satellite Availability and PDOP

The number of available satellites and PDOP values are commonly used indicators to assess the theoretical availability of satellite systems on a global scale. In order to explore how this indicator affects the positioning performance of Galileo SPP when using different data sources compared to the GPS system, this section assesses the theoretical availability of Galileo on a global scale, including the number of available satellites and PDOP values, the calculations of which require the known satellite and receiver coordinates. Satellite positions can be generally derived from both broadcast and precise ephemeris, and the differences between them are several meters, which can be ignored for satellite availability and PDOP calculation. Therefore, all satellite positions in this section are directly derived from the precise ephemeris of Wuhan University (WHU). The surface of the Earth was subdivided into a 2° (latitude) × 5° (longitude) grid, and a virtual receiver is considered to be placed at the center of each grid with zero altitudes. For the purpose of comparison, the number of available satellites and the PDOP of GPS are also presented.

**Figure 2:**
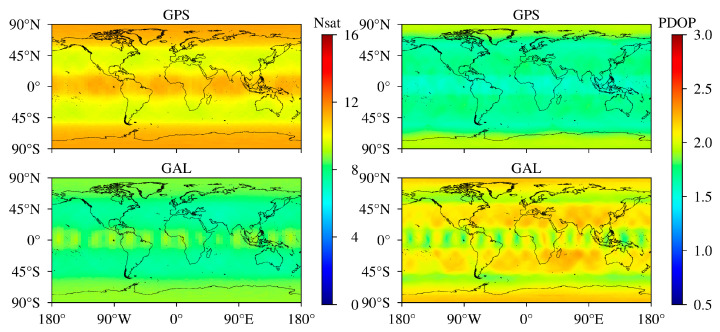
Average number of visible satellites (**left**) and PDOP (**right**) for GPS (**top**) and Galileo (**bottom**). Number of satellites and PDOP with an elevation mask of 7°.

The average values of the number of visible satellites and PDOP over a day (DOY 32/2021) for Galileo and GPS with an elevation mask of 7° are shown in Figure 2. Compared with those in mid-latitude regions, it is worth noting that there are more visible satellites in high- and low-latitude regions. Specifically, there are 7.0–8.8 and 9.4–11.1 visible satellites for Galileo and GPS in the mid-latitude region. For low- and high-latitude regions, 7.3–9.3 and 9.7–11.6 satellites can be observed for Galileo and GPS, respectively. As can be seen, the number of visible satellites in the current Galileo constellation is actually smaller than that of GPS around the world. PDOP is closely related to the number of visible satellites and their spatial distribution. In general, more visible satellites mean smaller PDOP values, and it is easy to draw this conclusion from the comparison of Galileo and GPS. However, there are still exceptions, such as for high-latitude regions, since the elevation angles of satellites in this region are generally small and more visible satellites do not have optimized PDOP, indicating poor geometry of the satellites. It can be seen that Galileo PDOP ranges from 1.7 to 2.3 around the world, while GPS PDOP is less than 2.0. 

**Figure 3:**
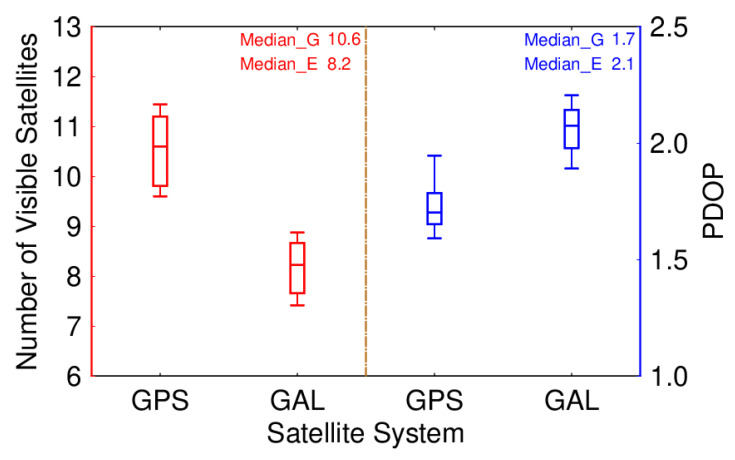
Distribution of number of visible satellites (**left**) and PDOP (**right**) for GPS and Galileo.

Moreover, the distribution of the number of visible satellites and PDOP values for Galileo and GPS, which is described with a boxplot, is depicted in Figure 3. G represents GPS, while E represents Galileo. It can be clearly seen from the range of the number of visible satellites and PDOP values. For Galileo, the number of visible satellites has a range of 7.4–8.9, while PDOP values range from 1.9 to 2.2, while for GPS, they are 9.6–11.4 and 1.6–1.9, respectively. 

## 4. Results Validation and Discussion

### 4.1. The Completeness of Galileo Navigation Message Records

First, we define an Empirical Completeness Rate (ECR) to describe the completeness of Galileo navigation message records, which is

(11) $$f_{\mathrm{ECR}}=\frac{N_{\mathrm{appeared}\;}}{N_{\mathrm{total}\;}}$$

where $N_{\mathrm{appeared}}$ denotes the number of navigation message records that appeared in RINEX files for all Galileo satellites; $N_{\mathrm{total}}$ denotes the total number of navigation message records for all Galileo satellites in theory; $f_{\mathrm{ECR}}$ is the ratio of $N_{\mathrm{appeared}}$ and $N_{\mathrm{total}}$ to indicate the completeness of Galileo’s broadcast ephemeris. As is known, the interval of Galileo navigation message records is 10 min. Taking FNAV as an example, we can obtain $N_{\mathrm{total}}=144\times 24=3456\;$ from 1-day Galileo navigation message records, where 24 is the number of Galileo satellites. If some navigation message records are lost, ECR will be less than 100%. 

**Figure 4:**
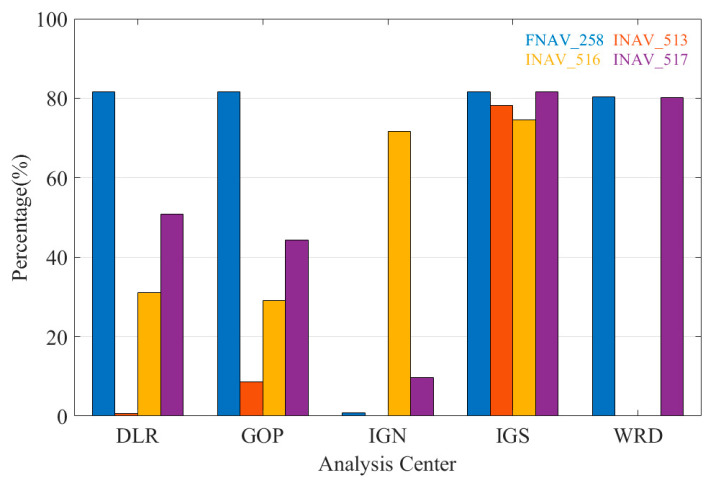
The completeness of merged Galileo broadcast ephemeris from different agencies.

The merged multi-GNSS navigation files in RINEX 3.xx format from IGS, IGN, DLR, GOP, and WRD are selected for validation. Figure 4 illustrates the completeness of INAV and FNAV in a total of 50 days of Galileo broadcast ephemeris provided by the four agencies from 1 February 2021. The different colored bars correspond to the second word of the fifth record (“Data Sources,” i.e., generally 258, 513, 516, 517) in the Galileo broadcast ephemeris, which indicates the different data sources of the broadcast ephemeris. To be concise, we use FNAV_258, INAV_513, INAV_516, and INAV_517 to represent different data sources of Galileo navigation message records. It is clear that the broadcast ephemeris provided by institutions other than IGS have varying degrees of lack of completeness for INAV and FNAV. IGS provides the best completeness in all aspects (ECR > 70%), while IGN supplies the worst one. Specifically, for the INAV_516 dataset provided by IGN, its completeness rate is relatively higher compared to other data sources, but it stands at only 71.6%. The completeness of DLR and GOP is similar in FNAV_258 and INAV_517; however, for INAV_513 and INAV_516, each has its own pros and cons. What is more, ephemeris provided by WRD has the same high availability in FNAV_258 and INAV_517 as IGS, despite lacking data in INAV_513 and INAV_516. To sum up, from the perspective of institutional performance, it is recommended that users choose IGS broadcast ephemeris when it comes to Galileo pseudorange-based point positioning in post-processing mode. 

Table 3 presents the information quantity of FNAV and INAV in the Galileo broadcast ephemeris provided by the IGS from 1 February 2021 for a total of 50 days. Among the 50-day information count, FNAV_258 has the highest quantity, while INAV_517 has slightly fewer records. INAV_513 and INAV_516 have comparatively lower quantities, with INAV_516 having the least. The proportions of FNAV_258, INAV_513, INAV_516, and INAV_517 during the statistical period are 25.83%, 24.76%, 23.61%, and 25.80%, respectively. Therefore, it is concluded that FNAV_258 and INAV_517 exhibit better data completeness, whereas INAV_513 and INAV_516 show poorer data completeness, with FNAV_258 having the highest data completeness and INAV_516 the lowest.

**Figure 5:**
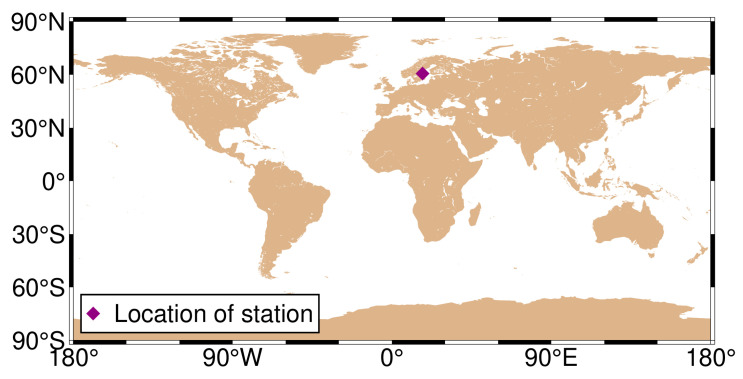
Geographical distribution of the MAR7 station.

**Figure 6:**
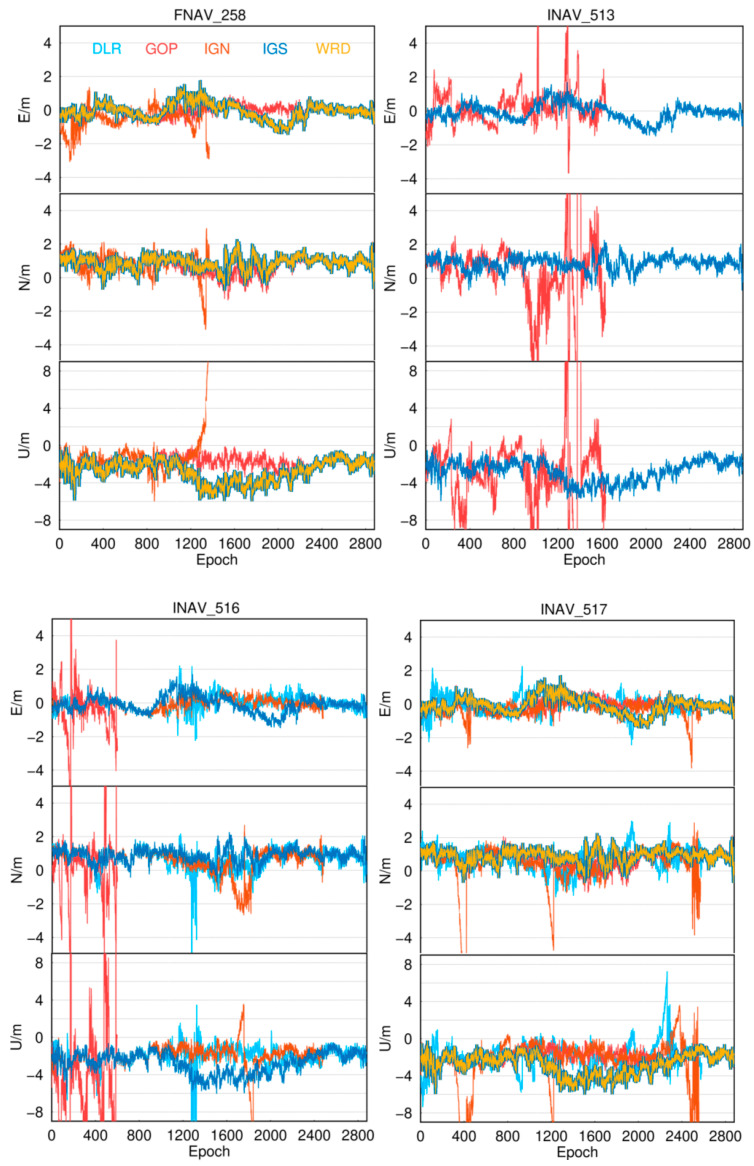
The positioning error sequences for the MAR7 station were obtained using navigation files from different analysis centers and different data sources from the Galileo satellite system.

We chose the MAR7 station to visually demonstrate positioning results from different institutions, using this station as an example. The MAR7 station is located at (17.2585° E, 60.5951° N), the GNSS receiver type is TRIMBLE ALLOY, and the antenna type is LEIAR25.R3 LEIT. The geographic distribution of the MAR7 stations is shown in Figure 5. The ECR and quality of navigation files provided by different institutions are not uniform. Error sequences obtained by employing navigation files from various institutions and utilizing different data sources for the MAR7 station are depicted in Figure 6. The figure demonstrates that, with the support of navigation files supplied by IGS, the best solution integrity and accuracy quality are attainable, with horizontal errors less than 2 m and vertical errors less than 5 m. Although the solutions obtained using navigation files from WRD are similar for FNAV_258 and INAV_517, they exhibit gaps for INAV_513 and INAV_516. Therefore, when conducting Galileo pseudorange-based point positioning in post-processing mode, opting for IGS broadcast ephemeris often yields enhanced integrity and accuracy.

### 4.2. Galileo Single-Frequency SPP (E1, E5a, and E5b)

**Figure 7:**
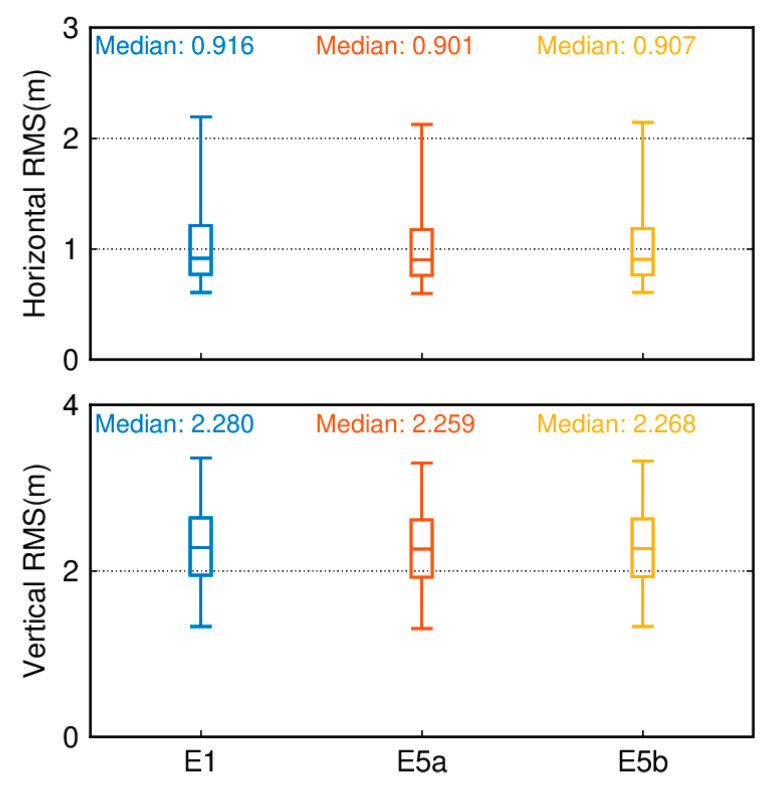
Boxplot of the position accuracy of Galileo single-frequency SPP in the E1, E5a, and E5b frequency bands.

We analyzed the overall positioning accuracy of these 137 tracking stations. Figure 7 illustrates the quartiles of positioning errors for Galileo single-frequency SPP in the E1, E5a, and E5b frequency bands with an elevation cutoff of 7°. The lower edge of the box represents the 5th percentile, the upper edge represents the 95th percentile, and the box’s lower, middle, and upper parts represent the 25th, 50th, and 75th percentiles, respectively. In terms of the horizontal component, the E5a band exhibits the highest positioning accuracy compared to others, with a minimum median root mean square (RMS) of 0.901 m, followed by the E5b band at 0.907 m and the E1 band with the poorest performance at 0.916 m. Regarding the vertical component, the positioning accuracy still follows the order of single-frequency E5a being superior to E5b, which is superior to E1, with median RMS values of 2.259/2.268/2.280 m, respectively.

**Figure 8:**
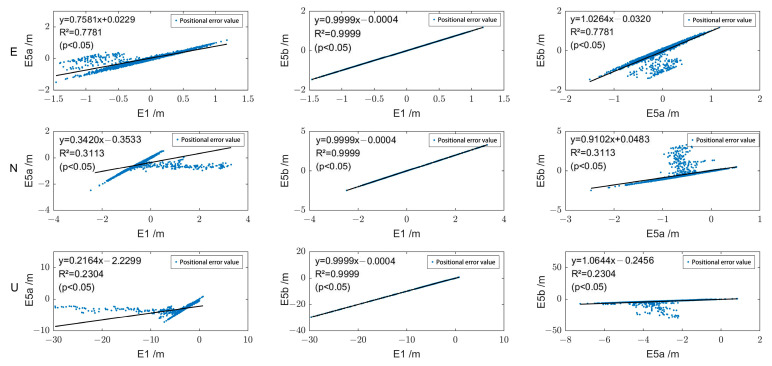
The correlation of positioning error sequences of Galileo single-frequency SPP using E1, E5a, and E5b at station ABPO.

**Figure 9:**
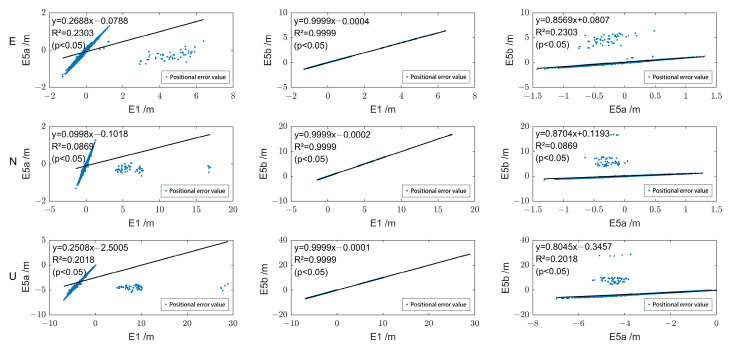
The correlation of positioning error sequences of Galileo single-frequency SPP using E1, E5a, and E5b at station CIBG.

Figure 8 and Figure 9 illustrate the correlation of the positioning error sequences of Galileo single-frequency SPP using E1, E5a, and E5b, taking stations ABPO and CIBG as examples. It can be observed that in the E, N, and U directions, the correlation between E1 and E5b is the highest, with correlation coefficients of 0.99. In the E, N, and U directions, the correlation coefficients between E1 and E5a, as well as between E5a and E5b, are identical. For station ABPO, the correlation coefficients are 0.88 (E), 0.56 (N), and 0.48 (U), while for station CIBG, they are 0.48 (E), 0.29 (N), and 0.45 (U).

### 4.3. Galileo Dual-Frequency SPP (E1/E5a, and E1/E5b)

**Figure 10:**
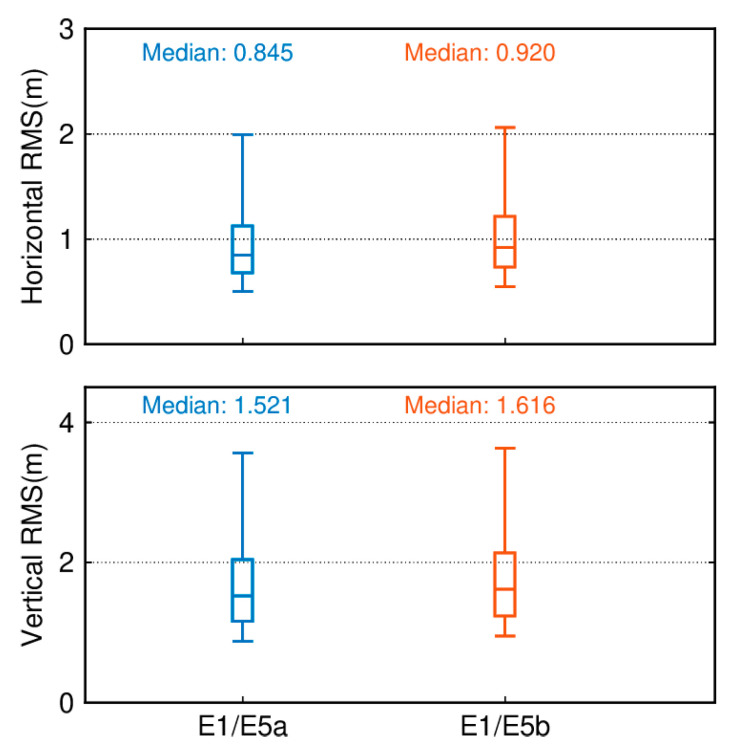
Boxplot of the position accuracy of Galileo dual-frequency SPP in the E1/E5a and E1/E5b frequency bands.

Figure 10 illustrates the quartiles of positioning accuracy for Galileo dual-frequency SPP in the E1/E5a and E1/E5b frequency bands with an elevation cutoff of 7°. In terms of the horizontal component, the E1/E5a band demonstrates higher positioning accuracy with an RMS of 0.845 m, followed by the E1/E5b band at 0.920 m. Regarding the vertical component, the positioning accuracy remains superior in the E1/E5a band compared to the E1/E5b band, with a median RMS of 1.521 and 1.616 m, respectively.

**Figure 11:**
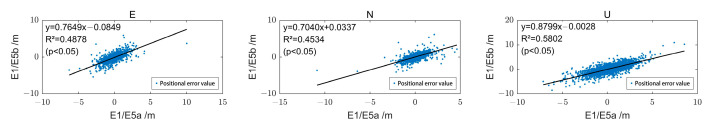
The correlation of positioning error sequences of Galileo dual-frequency SPP using E1/E5a and E1/E5b at station ABPO.

**Figure 12:**
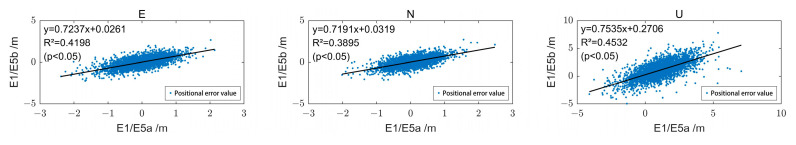
The correlation of positioning error sequences of Galileo dual-frequency SPP using E1/E5a and E1/E5b at station CIBG.

Figure 11 and Figure 12 depict the correlation of the positioning error sequences of Galileo dual-frequency SPP using E1/E5a and E1/E5b, with stations ABPO and CIBG as examples. In contrast to single-frequency observations, both stations ABPO and CIBG exhibit the highest correlation in the U direction, with values of 0.76 and 0.67, respectively, and the lowest correlation in the N direction, with values of 0.67 and 0.62, respectively. The correlation coefficients in the E, N, and U directions for both stations are relatively close. Combined with Figure 8, Figure 9, Figure 11 and Figure 12, it is evident that dual-frequency observations effectively reduce the data dispersion.

### 4.4. Performance of Galileo Single-Frequency SPP with Different Data Sources

**Figure 13:**
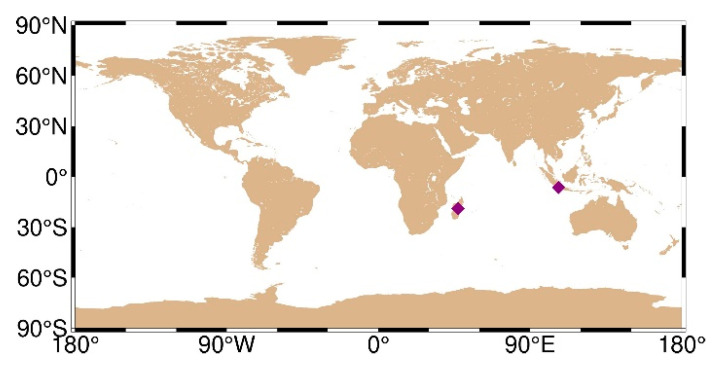
Geographical distribution of the ABPO (left purple diamond-shaped dots) station and CIBG (right purple diamond-shaped dots) station.

**Figure 14:**
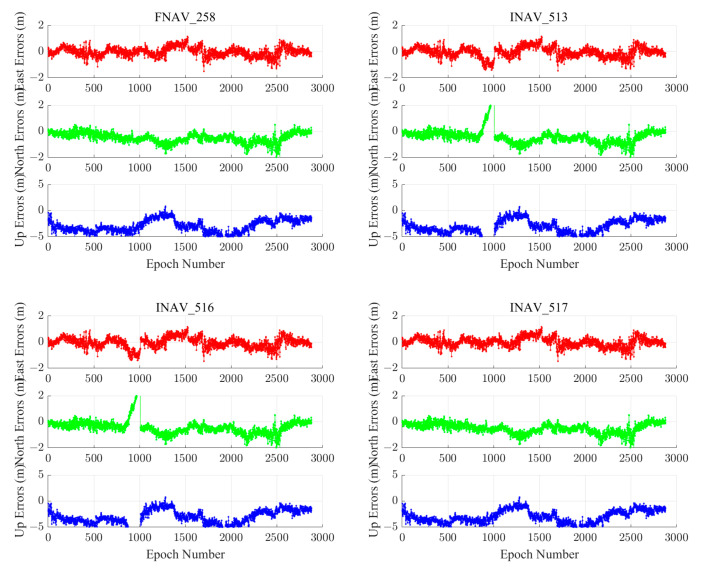
Single-frequency positioning error sequences of the ABPO station using different types of navigation information data sources from the Galileo satellite system.

**Figure 15:**
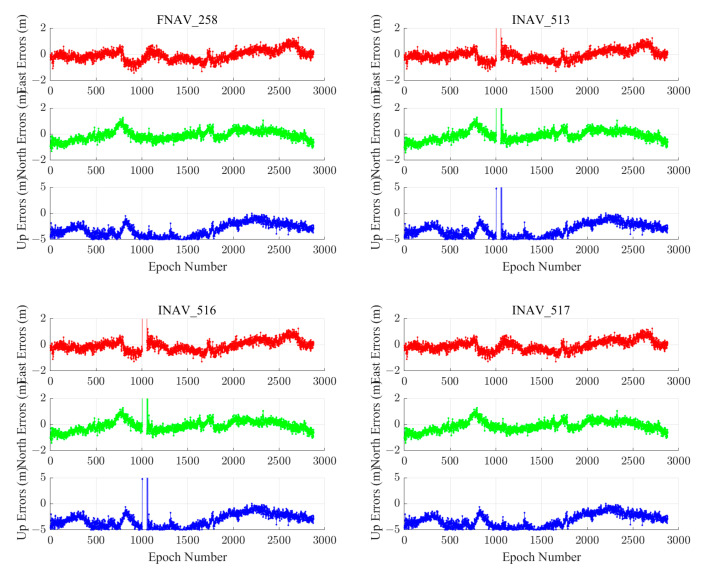
The positioning error sequences of the CIBG station were obtained using different types of navigation information data sources from the Galileo satellite system.

We chose the ABPO and CIBG stations to visually demonstrate the error sequence distributions using FNAV_258, INAV_513, INAV_516, and INAV_517. The ABPO station is located at (47.2292° E, 19.0183° S), the GNSS receiver type is SEPT POLARX5, and the antenna type is ASH701945G_M SCIT. The CIBG station is located at (106.8492° E, 6.4904° S), the GNSS receiver type is TRIMBLE NETR9, and the antenna type is LEIAR25.R4 NONE. The geographic distribution of the ABPO station and the CIBG station is shown in Figure 13. The error sequence plots for the ABPO and CIBG stations on DOY 32/2021 are depicted in Figure 14 and Figure 15, respectively. These plots illustrate the errors associated with broadcast ephemeris data obtained from different sources provided by the IGS. It is evident that better positioning results are achieved when using navigation information data sources FNAV_258 and INAV_517, with positioning errors within 2 m in the east, 2 m in the north, and 5 m in the up directions. However, the positioning accuracy is poorer when using the navigation information data sources INAV_513 and INAV_516. For the ABPO station, although the positioning error in the E direction is similar to that of FNAV_258 and INAV_517 when using INAV_513 and INAV_516, there is divergence in the results in the N and U directions. As for the CIBG station, the use of INAV_513 and INAV_516 results in varying degrees of divergence in positioning results in the E, N, and U directions.

**Figure 16:**
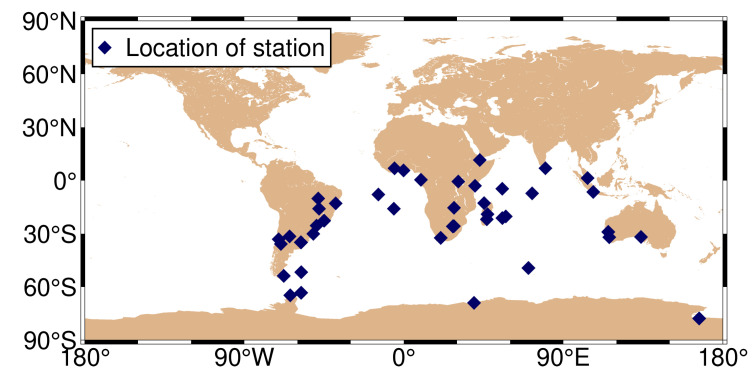
Geographical distribution of the 52 MGEX stations with significant differences in positioning results when using different data sources.

The geographic distribution of the stations with significant differences in positioning results when using different data sources is depicted in Figure 16, where the station locations are represented by blue squares. It is noteworthy that there are a higher number of stations with divergent errors in the southern hemisphere compared to the northern hemisphere. Specifically, the occurrences of stations at low-, mid-, and high-latitudes are 24, 25, and 3, respectively, accounting for 46.15%, 48.08%, and 5.77% of the stations. Stations located at middle to low latitudes make up 94.23% of the total, indicating that the Galileo satellite navigation system generally provides better positioning accuracy and stability in the high-latitude regions of the northern hemisphere. 

Based on the corresponding date of the station (DOY 32/2021), a statistical analysis was conducted on the navigation files provided by IGS. $N_{258}$ represents the number of navigation message records for FNAV_258 in all Galileo satellites’ RINEX files, which is 2604. Similarly, $N_{513}$, $N_{516}$, and $N_{517}$ represent the number of navigation message records for INAV_513, INAV_516, and INAV_517, with corresponding quantities of 2581, 2581, and 2601, respectively. The absence of navigation information often results in the divergence of positioning results, and this is observed with fewer navigation message records for INAV_513 and INAV_516, which experienced divergence in positioning results. Based on the content shown in Table 4, when different data sources appear for the same satellite, the differences between FNAV_258 and INAV_517 are relatively small, but they are significantly larger when compared to INAV_513 and INAV_516. It is evident that the navigation information for the E18 satellite in INAV_513 and INAV_516 is severely missing, which could be a cause of the divergence in the positioning solution. Specifically, in terms of time, the error interval occurred between 900 and 1000 epochs, which translates to UTC time between 7:30 and 8:30. Within this time interval, the navigation message record counts for FNAV_258 and INAV_517 were 125 each, while INAV_513 and INAV_516 had 123 each. By comparing the results, it was discovered that the navigation information for PRN number E18 of INAV_513 and INAV_516 satellites was missing, resulting in the divergence of the positioning results. For the ABPO station, the divergence occurred in the E and U directions, whereas for the CIBG station, it was observed in the E, N, and U directions. 

**Figure 17:**
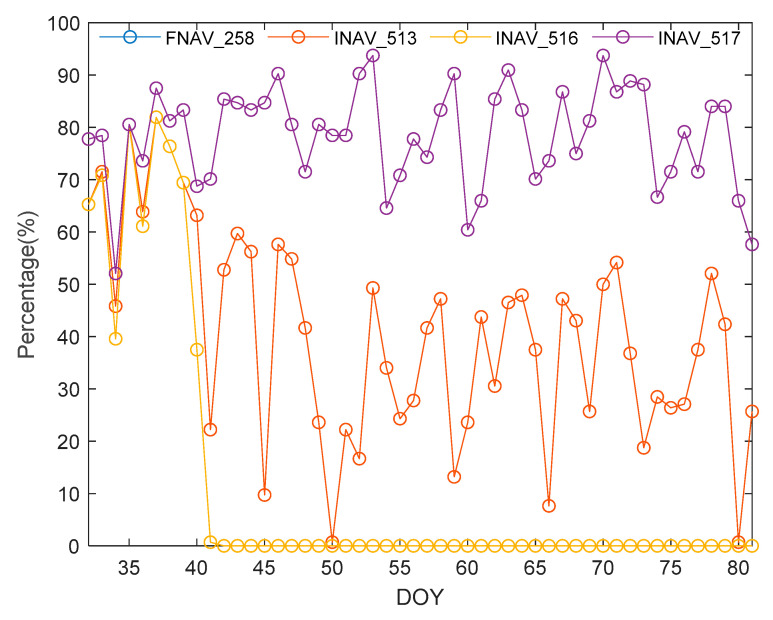
Ephemeris completeness rate of satellite E18 from different data sources.

E18 is in a moderately elliptical orbit (eccentricity of 0.162). The E18 satellite of the Galileo satellite navigation system was subjected to ECR calculation during the research period, as shown in Figure 17. Due to the high similarity in completeness between the navigation information of FNAV_258 and INAV_517, it appears that there is missing data for FNAV_258. It is evident from the results that the navigation information of the E18 satellite in FNAV_258 and INAV_517 is relatively complete, with an average completeness rate close to 78.56% during the study period. However, the completeness of INAV_513 and INAV_516 is relatively poor, with completeness rates of 40.54% and 11.67%, respectively. Consequently, when using the Galileo satellite navigation system for position determination during this time period, it is observed that there is a scarcity of available satellites, and using the E18 satellite may lead to divergence in the positioning results.

**Figure 18:**
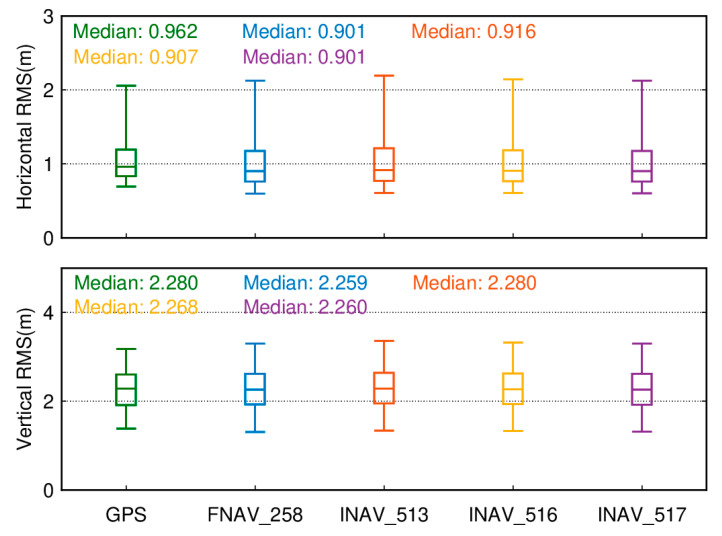
Boxplot of the RMS for single-frequency SPP results of the GPS system and the Galileo system with different data sources.

Figure 18 presents box plots of the RMS values for SPP results of 137 tracking stations based on the GPS system and different data sources for the Galileo system. The upper part of Figure 18 displays a boxplot of the RMS of positioning errors in the horizontal direction for Galileo single-frequency SPP using different data sources and GPS single-frequency SPP over 50 days. The lower part of Figure 18 displays a boxplot of the RMS of positioning errors in the vertical direction for Galileo single-frequency SPP using different data sources and GPS single-frequency SPP over 50 days. The lower edge of the box represents the 5th percentile, the upper edge represents the 95th percentile, and the box’s lower, middle, and upper parts represent the 25th, 50th, and 75th percentiles, respectively. The positioning results are obtained by averaging measurements taken over 50 days from day 032 to day 081 of the year 2021. In the horizontal direction, it can be observed that using FNAV_258 and INAV_517 yields better horizontal positioning accuracy, with RMS values of 0.901 m and 0.901 m, respectively. The horizontal positioning accuracy using INAV_513 and INAV_516 is slightly lower than that of the former but better than that of the GPS system. Similar conclusions can be drawn from the vertical perspective, where employing FNAV_258 and INAV_517 provides better vertical positioning accuracy, with RMS values of 2.259 m and 2.260 m, respectively. Similarly, the vertical positioning accuracy using INAV_513 and INAV_516 is slightly lower than that of the former but superior to the GPS system’s vertical positioning accuracy. Although the horizontal and vertical positioning accuracy of the GPS system is slightly lower compared to the Galileo system, its 95th percentile RMS values are lower than those of the Galileo satellite system. As a result, the error fluctuations are smaller, leading to a smoother sequence of positioning results.

**Figure 19:**
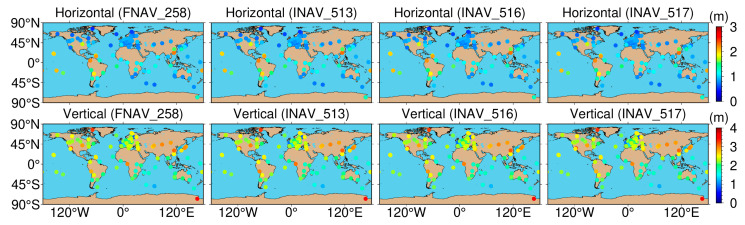
Geographical distribution of single-frequency positioning accuracy in both horizontal and vertical sections for 137 tracking stations using Galileo with different data sources.

Figure 19 presents the geographical distribution of positioning accuracy for 137 tracking stations using Galileo single-frequency SPP with different data sources. In the figure, white indicates values surpassing the upper limit of the ribbon chart. As depicted, the geographical distribution of positioning accuracy remains largely consistent across various data sources. Regarding the horizontal component, tracking station accuracy exhibits a pronounced dependence on latitude. Stations situated in the mid to high latitudes of North America and Europe display a RMS value of approximately 0.8–1.2 m. However, in mid-latitudes and low-latitudes, the RMS values for tracking stations increase to around 2 m, and in low-latitude regions of South America, the RMS values almost reach 3 m. In the vertical component, tracking stations in Europe demonstrate the highest positioning accuracy, whereas those in Asia exhibit the lowest accuracy. Stations located in the Arctic and Antarctic regions display the poorest positioning accuracy, with RMS values reaching 4 m. 

### 4.5. Performance of Galileo Dual-Frequency SPP with Different Data Sources

Single-frequency pseudorange positioning is susceptible to signal errors, such as atmospheric delay and clock bias errors, leading to limited positioning accuracy. Dual-frequency SPP is a method that uses signals from two different frequencies for positioning, and this method has higher accuracy and better resistance to errors like atmospheric delay compared to single-frequency SPP. 

**Figure 20:**
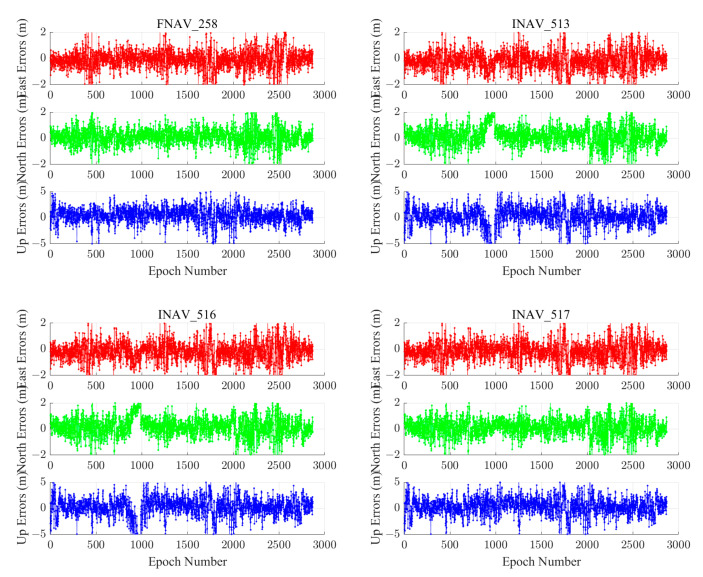
Dual-frequency positioning error sequences of the ABPO station using different types of navigation information data sources from the Galileo satellite system.

**Figure 21:**
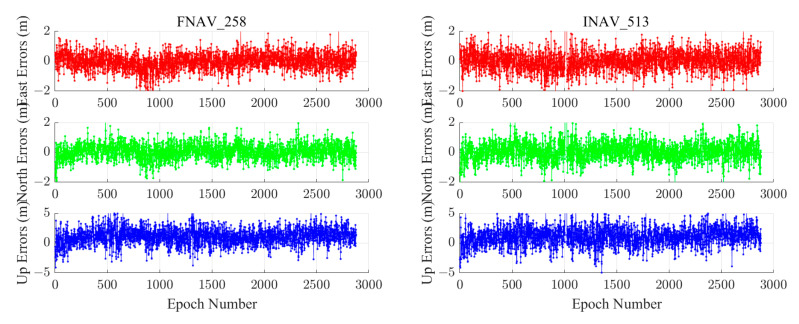

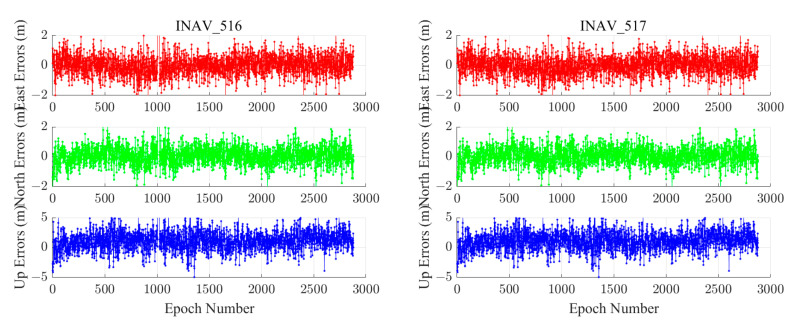
Dual-frequency positioning error sequences of the CIBG station using different types of navigation information data sources from the Galileo satellite system.

Figure 20 and Figure 21 illustrate the dual-frequency positioning errors for two measurement stations. The single-frequency systematic errors in the E and N directions are relatively small. The addition of dual-frequency observations introduces observation noise, resulting in a slight reduction in accuracy. While a phenomenon of lower precision reduction is observed, it optimizes the divergence phenomenon in the single-frequency solution process. In the vertical direction, the inclusion of dual-frequency observations reduces the vertical error RMS from 3–4 m to 1–2 m, significantly enhancing vertical positioning accuracy. For dual-frequency pseudorange single-point positioning reliant on the Galileo satellite system, the solutions obtained using FNAV_258 and INAV_517 remain superior to INAV_513 and INAV_516, while the solutions from INAV_513 and INAV_516 are both inferior and comparable. This phenomenon closely resembles that observed in the single-frequency Galileo satellite system pseudorange SPP.

**Figure 22:**
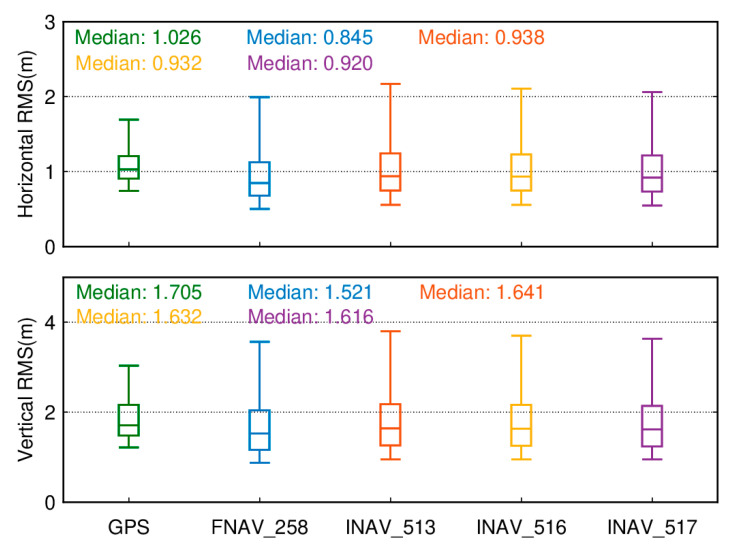
Boxplots of positioning accuracy for dual-frequency ionosphere-free combined SPP within the GPS system and the Galileo system using different data sources.

The distinction between dual-frequency ionosphere-free combined SPP and single-frequency SPP lies in the fact that single-frequency SPP employs a priori models to mitigate ionospheric effects. In contrast, dual-frequency SPP utilizes an ionosphere-free combination to eliminate ionospheric errors. Figure 22 depicts boxplots of positioning accuracy for dual-frequency ionosphere-free combined SPP within the GPS system and the Galileo system using different data sources. In terms of positioning accuracy, the dual-frequency ionosphere-free combined SPP within the Galileo system demonstrates an advantage over the GPS system, though the GPS system’s 95th percentile RMS is notably lower than that of the Galileo system, indicating smoother solution results for the GPS system. Regarding horizontal components, among the 137 tracking stations utilizing different data sources within the Galileo dual-frequency ionosphere-free combined SPP, the median RMS values are 0.845 m (FNAV_258), 0.938 m (INAV_513), 0.932 m (INAV_516), and 0.920 m (INAV_517). In comparison to single-frequency solutions, except for FNAV_258, all medians experience slight increases. However, noticeable reductions are observed in the 95th percentile RMS, effectively diminishing dispersion levels. In the vertical component, while the addition of dual-frequency observations leads to a slight increase in the 95th percentile RMS values, vertical positioning accuracy significantly improves. The RMS values decrease from over 2 m to below 2 m, particularly pronounced for FNAV_258, where the median drops from 2.259 m to 1.521 m. Incorporating dual-frequency observations effectively reduces dispersion and enhances vertical positioning accuracy.

**Figure 23:**
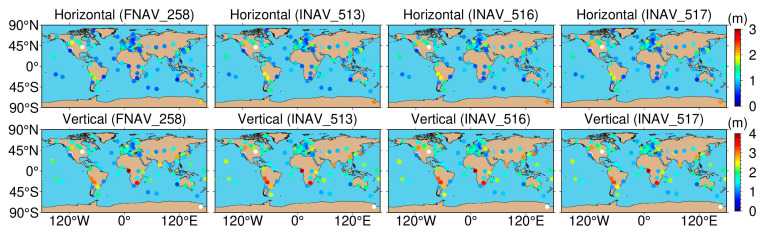
Geographical distribution of dual-frequency positioning accuracy in both horizontal and vertical sections for 137 tracking stations using Galileo with different data sources.

Figure 23 depicts the geographical distribution of positioning accuracy for 137 tracking stations using Galileo dual-frequency SPP with different data sources. As shown in the figure, the geographical distribution of positioning accuracy remains largely consistent across various data sources. In the horizontal component, compared to single-frequency, there is a slight decrease in the positioning accuracy of some tracking stations in the mid- to high-latitude regions for dual-frequency. However, tracking stations located in low-latitude regions exhibit a significant improvement in positioning accuracy. In the vertical component, except for a few tracking stations in low-latitude regions of South America and Africa, dual-frequency positioning accuracy experiences a noticeable enhancement compared to single-frequency. Stations in North America and Europe still maintain superior accuracy compared to the Asian region.

## 5. Conclusions

The Galileo system is Europe’s own global navigation satellite system. It is a system that provides high-precision, committed global positioning services for civilian control and can interoperate with the GPS and GLONASS global navigation and positioning systems. In this study, 137 tracking stations were selected to analyze the theory and positioning performance of SPP, leading to the following conclusions:

(1) IGS provides the highest completeness in all aspects (ECR > 70%), while IGN offers the lowest completeness. Users selecting IGS broadcast ephemeris for Galileo SPP tend to achieve better solution results in post-processing mode. Additionally, within the IGS broadcast ephemeris, FNAV_258 and INAV_517 exhibit relatively high and similar data completeness, while INAV_513 and INAV_516 show slightly lower and similar data completeness. Combining the analysis of SPP performance across 137 global stations, higher data completeness corresponds to higher positioning accuracy.
(2) The global satellite visibility and PDOP values for GPS and Galileo are similar, exhibiting a symmetric pattern between the northern and southern hemispheres. Compared to mid-latitude regions, both high-latitude and low-latitude areas have more visible satellites. Although GPS demonstrates better global satellite visibility and PDOP values, analysis of SPP performance across 137 global stations indicates that the positioning accuracy of Galileo SPP using different data sources surpasses that of GPS. However, GPS demonstrates lower 95th percentile RMS values, providing the advantage of smaller error fluctuations and smoother positioning result sequences. Furthermore, the introduction of dual-frequency observations effectively reduces data dispersion and enhances vertical positioning accuracy.

The results of this study clearly demonstrate the Galileo system’s SPP positioning accuracy using different data sources. In addition to understanding the integrity of navigation information data provided by different institutions, this study also provides references for selecting the optimal data source for Galileo SPP computation.

## Figures and Tables

**Table 1:** Galileo navigation message orbit parameters.

Nav.Record	Description
Broadcast orbit-1	Issue of Data of the nav batch
	Crs (m)
	Delta n (radians/s)
	M0 (radians)
Broadcast orbit-2	Cuc (radians)
	e Eccentricity
	Cus (radians)
	sqrt(a) (sqrt(m))
Broadcast orbit-3	Toe Time of Ephemeris (sec of GAL week)
	Cic (radians)
	OMEGA0 (radians)
	Cis (radians)
Broadcast orbit-4	i0 (radians)
	Crc (m)
	omega (radians)
	OMEGA DOT (radians/s)
Broadcast orbit-5	IDOT (radians/s)
	Data sources
	GAL Week
Broadcast orbit-6	Signal in space accuracy (m)
	Satellite health status
	BGD E5a/E1 (s)
	BGD E5b/E1 (s)
Broadcast orbit-7	Transmission time of message

**Table 2:** The strategies of SPP processing.

Items	Strategies
Number of tracking stations	137
Number of satellites	Galileo (30), GPS (32)
Signal selection	Galileo (E1, E5a, E5b), GPS (L1, L2)
Sampling rate	30 s
Satellite elevation cutoff	7°
Weight of observation value	Prior standard deviation of measurement error
Tropospheric delay	Saastamoinen delay model
Ionospheric delay	Single frequency and IF combination

**Table 3:** Statistics of different data sources for broadcast ephemeris provided by IGS.

DOY	Data Sources			
	FNAV_258	INAV_513	INAV_516	INAV_517
32–41	28,038	27,630	27,276	27,996
42–51	29,324	27,922	26,217	29,265
52–61	29,057	27,777	26,562	29,020
62–71	27,398	26,030	24,370	27,361
72–81	27,262	25,852	24,531	27,237
ECR	81.64%	78.25%	74.63%	81.53%

**Table 4:** Differences in the quantity of broadcast ephemeris from different data sources provided by IGS.

PRN	Data Sources (DOY 32/2021)			
	FNAV_258	INAV_513	INAV_516	INAV_517
E07	105	106	106	106
E11	129	128	128	128
E12	115	114	114	114
E14	105	103	103	105
E18	112	94	94	112
E24	123	122	122	122
E25	100	99	99	99

## Data Availability

The GNSS raw observation data are available at ftps://gdc.cddis.eosdis.nasa.gov/pub/gnss/data/daily/ (accessed on 19 July 2023). The broadcast ephemeris data are available at ftps://gdc.cddis.eosdis.nasa.gov/pub/gnss/data/daily/2021/brdc/ (accessed on 19 July 2023).
